# Cases of Sarcomatous Tumor

**Published:** 1876-11

**Authors:** J. Ewing Mears


### ARTICLE II.

### Cases of Sarcomatous Tumor.

BY J. EWING MEARS, M. D.,

The two cases of sarcomatous tumor, to which I beg to direct the attention of the Fellows of the College this evening, presented in their external appearances and in other symptoms such sufficiently well marked resemblances as to induce me to offer them for consideration together. Differing as to point of departure, they were brought into close relation in the process of development by the involvement of similar structures.

Case 1st.—The first case was one of peripheral osteosarcoma, taking its origin in the periosteum of the lower jaw, in a boy twelve years of age, a resident of Delaware, who was brought to my clinic at the Pennsylvania College of Dental Surgery, in the month of October, 1873. The following history with regard to the cause of the tumor was obtained at that time: In the month of July preceding, whilst in bathing, he received a severe blow upon the left of the face over the region of the lower jaw, caused by coming in contact with the head of one of his comrades as he sprang into the water from the pier. At the time of the receipt of the injury he experienced considerable pain, and soon after, swelling supervened. The soreness in the part gradually diminished and finally disappeared, leaving however, a slight enlargement. This was so slight as to produce no deformity, and in a few weeks the accident and its results were dismissed from mind.

During the course of the summer, attention was attracted to a swelling of the face, which was attributed to a suspected decayed tooth which had been attacked by cold. The usual remedies employed in such cases were applied without causing the disappearance of the swelling, and the boy was placed under the care of a physician in the town in which he resided. At first the physician was disposed to regard the swelling as due to the cause ascribed by the members of the family, and directed treatment accordingly. Failing to obtain the results expected from the treatment, he was led to make a more careful and extended examination, and arrived at the conclusion that the case was of a more serious nature; he then advised that the patient should be brought to the city and placed under the care of a surgeon.

On examination the left side of the face was found very much enlarged, the swelling occupying more particularly the region of the lower jaw, the greater portion of the body, the angle and ramus. To the touch it was hard, dense, and inelastic, and firmly attached to the bone. The overlying tissues were freely movable and yielding. The finger introduced between the cheek and the teeth into the buccal space could distinctly trace the attachment of the growth to the lower jaw. The external surface of the upper jaw was normal and the canine fossa intact, showing the antrum to be free from disease. There was almost entire occlusion of the jaws, the patient being able by great effort to separate them to the extent of but one quarter of an inch. In order to examine the internal surface of the lower jaw it was necessary to give the patient ether and forcibly separate the jaws by a lever, when the internal surface was found normal and not in any way affected by the tumor. The introduction of the exploring needle into the mass from the inside was followed by an escape of blood.

At no time during the development of the growth had pain been a prominent symptom. The increase in size had been very rapid, and the occlusion of the jaws compelled the patient to confine himself to the ingestion of liquid food. His general health was not notably impaired. Having determined the nature of the growth and its position, I advised its immediate removal, which was acceded to, and the operation was performed at the residence of the patient, Drs. R. R. Taylor and Parish, of this city, and Dr. Harmon, U. S. Navy, being present and assisting.

Owing to the closure of the jaws, I found it impracticable to effect removal by an internal incision, beginning just below the lobule of the ear, carrying it downwards to the angle of the jaw, along the posterior border of the ramus, and thence forward along and slightly posterior to the inferior border to a point within one inch of the symphysis. The facial artery was secured at the anterior inferior angle of the masseter muscle, where it mounts upon the jaw, and divided. Dissecting the skin and superficial fascia upwards to some distance, a freely movable flap was formed, which, on being raised, exposed the upper portion of the tumor. In order to reach its attachments below, it was found necessary to detach the superficial structures to some extent into the region of the neck posteriorly. The masseter muscle at its insertion was incorporated in the tumor; above, to one-third of its extent, it was free, and on being divided, retracted to its points of origin. Having fully exposed the tumor, an effort was made to separate it from the jaw by the knife; but this was found impossible, and recourse was had to the chisel, by which it was detached, removing with it the external plate of the bone softened and disintegrated to the extent of attachment of the tumor; posteriorly, the growth passed over the border of the ramus, and penetrated the deeper structures of the neck, overlying the external carotid artery; this portion was removed by careful dissection, and the rough spiculated surface of the bone was thoroughly scraped by the chisel.

Having ascertained by a careful examination of the part that all of the morbid structure was removed, the wound was closed by six iron-wire sutures, a compress and bandage were applied, and the patient was placed in bed. At the expiration of three weeks the greater portion of the incision had united, a sinus remaining, through which small spicules of bone were discharged; the boy was sent to the country, and in the course of two months the sinus closed; since then he has enjoyed uninterrupted good health, and has been able to resume his studies in school. The occlusion of the jaws was at once relieved by the operation, and they can now be separated to the normal extent.

The sarcomatous tumor removed from the jaw of the boy presents the microscopical appearances of a small round-celled structure with a delicate, finely granular, occasionally fibrillated intercellular tissue. It also contains numerous striated muscular fibres; many of them being found in the several stages of fatty degeneration and atrophy. But there are also some muscular fibres, which are of great interest, showing, what I believe to be, intra-muscular sarcoma. These fibres exhibit at many points such a decided increase in the number of nuclei of the muscular substance by segmentation, while at the same time the general appearance of the cells thus formed, as well as their reaction under various chemical re-agents, is such as to leave scarcely any doubt that the proliferation within the sarcolemma is of a sarcomatous nature, and not due to mere inflammatory processes.

These intra-muscular cells, still inclosed by the sarcolemma, have a round, occasionally an oval shape; a large nucleus which is readily stained with carmine and surrounded with a thin layer of protoplasm. In size, these cells equal those of the primary growth. They are not always uniformly disseminated throughout the length of the muscular fibres, but are often so aggregated as to completely fill out and distend the lumen of the cylinder. At a few points, where the growth of the sarcoma has been more rapid in the muscles than the invasion of the fibrillated structure of the perimysium, a parallel course is still maintained by the sarcomatous muscular fibres. The sarcous elements seem to passively disappear under the pressure of the newly formed cells without previously becoming granular or fatty. The transverse and longitudinal structures of the muscular fibres finally disappear entirely, and naught but cylinders filled with round cells, each separated by a fibrillated intercellular tissue, remain, *i. e.* the muscular substance has been transformed into young cellular connective tissue, and sarcomatous cells have been destroyed, and the muscular fibres there present a uniform sarcomatous tissue.

The majority of the muscular fibres seen in this tumor are undergoing passive changes, but in a few of them the active proliferation of the muscular nuclei, described above, has taken place, which are not primary but secondary changes induced by the sarcoma.

Sokolow was the first to demonstrate this active participation of the muscles in a spindle-celled sarcoma. I think this specimen conclusively proves that the muscular nuclei also can, and do, participate in the formation of the round-celled variety of sarcoma.

Case II.—The second case was one of sarcoma of the parotid gland of the right side, occurring in a patient who was admitted into the surgical wards of St. Mary's Hospital, in the summer of the year 1874, for a swelling which occupied the right side of his face.

He was 58 years of age, and a furrier by trade. He had enjoyed good health until two years ago, when he had a partial attack of paralysis involving the right side. This condition interfered somewhat with his work, but was not sufficient to compel him to abandon it. Three months prior to his admission he noticed a slight swelling upon the right side of the face, and at the same time experienced difficulty in opening his mouth to the usual extent. The swelling increased quite rapidly, and with its increase in size the occlusion of the jaws became more marked. A physician was consulted, who, regarding it as an inflammatory swelling, ordered sorbefacient applications. No benefit appeared to be derived from the treatment pursued, and the patient came to the hospital.

At the time of his admission the tumor had grown to quite a large size, and the occlusion of the jaws was nearly complete. An examination revealed the fact that the parotid gland and the masseter muscle were involved, and the jaw itself was supposed to be implicated. The removal of the growth having been determined upon, it was exposed by an incision extending from the zygoma downwards, in front of the ear, and along the border of the ramus and body of the jaw to within an inch and a half of the symphysis. The flaps formed by this incision were freely dissected upwards upon the cheek, and downwards upon the neck, and the tumor consisting of the parotid gland and the entire masseter muscle was removed. The facial and external carotid arteries were in turn ligated—the facial before, and the external carotid artery after division. The wound was closed by iron-wire sutures, and a compress and bandage were applied. Considerable suppuration ensued before the wound finally healed, which occurred four weeks after the operation.

The removal of so large a portion of the facial nerve as was necessary in performing the operation, intensified the facial paralysis which already existed. The patient is now in very good health. There is no evidence of any return of the disease. The occlusion of the jaws was entirely relieved by the operation.

The tumor removed from the parotid region is made up of large, round, and spindle-cells, together with a sparse, finely fibrillated, intercellular substance.

Certain portions of the growth reveal the presence of a glandular structure, also ducts lined with cylindrical epithelium, thus showing that a secondary invasion, if not primary involvement, of the parotid gland, exists. There is no excessive development of the epithelial lining, no formation of atypical epithelium, no ingrowing epithelial papillæ; but, on the other hand, the glandular parenchyma is widely separated, often compressed by an exuberant formation of sarcomatous elements in the surrounding connective tissue.

In the further consideration of these cases, it may be of interest to examine somewhat into certain features which seem to belong peculiarly to them. In general, it may be said that they illustrate the usual history of sarcomatous growths, and yet present such marked variations as to entitle them to a certain claim of distinction, in a clinical as well as a pathological point of view.

With regard to the latter, Dr. Bertolet has clearly described the very interesting condition which he observed to have occurred in both cases, that is, infiltration of the adjacent muscular tissue by the sarcomatous growth, an unusual feature, and one which belongs essentially to carcinomatous formations in marked contrast to sarcomas, which are usually encapsulated.

Clinically, the cases are of interest in the very rapid development of the tumors. In the first case, in which the cause was distinctly traced, a large size was attained in four months, and whilst under observation its growth was quite apparent; in the second case the increase in size was equally rapid. According to Billroth, (Surgical Pathology,) sarcomatous tumors of the parotid gland grow very slowly, and particularly so when they do not form until middle age.

The time of life at which the tumors appeared may be regarded as a departure from the rule which seems to govern them. Without regard to the tissues implicated, they are described as of rare occurrence between the ages of ten and twenty years, and as still rarer in old age. In the salivary glands they rarely occur after the fortieth year. In the cases under consideration, the disease manifested itself in early life, and somewhat after middle life.

As to the origin of the tumor of the jaw, it is quite evident that it was periosteal in character; the injury received developing the morbid action in the inner layer of the periosteum, an thus giving rise to a true periosteal sarcoma. The history, in the case of the tumor of the parotid gland, gives no information as to a cause, and it may be a question whether the involvement of the gland was primary or secondary.

The absence of any recurrence of the disease in the cicatrices and of any symptoms of metastatic formation up to this period of time after extirpation of the tumors, would permit a reasonably favorable prognosis to be made. This immunity was not to be expected in the second case, owing to the age of the patient; at his time of life speedy return of the diseases being the rule. The prognosis in the case of the boy was favorable as to his age, but unfavorable as to the character of the growth, periosteal sarcomas being regarded as quite malignant in their nature.

Extirpation is the only method of treatment to be pursued, and great care should be taken to remove all of the morbid tissue.—*Transactions of College of Physicians, June 7th*, 1876.

